# Multimodal Magnetic Resonance and Photoacoustic Imaging of Tumor-Specific Enzyme-Responsive Hybrid Nanoparticles for Oxygen Modulation

**DOI:** 10.3389/fbioe.2022.910902

**Published:** 2022-07-13

**Authors:** Maharajan Sivasubramanian, Chia-Hui Chu, Shih-Hsun Cheng, Nai-Tzu Chen, Chin-Tu Chen, Yao Chen Chuang, Hsia Yu, Yu-Lin Chen, Lun-De Liao, Leu-Wei Lo

**Affiliations:** ^1^ Institute of Biomedical Engineering and Nanomedicine, National Health Research Institutes, Zhunan, Taiwan; ^2^ Department of Radiology, The University of Chicago, Chicago, IL, United States; ^3^ College of Biopharmaceutical and Food Sciences, Institute of New Drug Development, China Medical University, Taichung, Taiwan

**Keywords:** manganese oxide nanoparticles, tumor-specific T1 contrast agent, hyaluronidase, magnetic resonance imaging, oxygen modulation, photoacoustic imaging

## Abstract

Multimodal imaging contrast agents for cancer that can not only perform diagnostic functions but also serve as tumor microenvironment–responsive biomaterials are encouraging. In this study, we report the design and fabrication of a novel enzyme-responsive T_1_ magnetic resonance imaging (MRI) contrast agent that can modulate oxygen in the tumor microenvironment via the catalytic conversion of H_2_O_2_ to O_2_. The T_1_ contrast agent is a core–shell nanoparticle that consists of manganese oxide and hyaluronic acid (HA)–conjugated mesoporous silica nanoparticle (HA-MnO@MSN). The salient features of the nanoparticle developed in this study are as follows: 1) HA serves as a targeting ligand for CD44-expressing cancer cells; 2) HA allows controlled access of water molecules to the MnO core via the digestion of enzyme hyaluronidase; 3) the generation of O_2_ bubbles in the tumor by consuming H_2_O_2_; and 4) the capability to increase the oxygen tension in the tumor. The *r*_1_ relaxivity of HA-MnO@MSN was measured to be 1.29 mM^−1^s^−1^ at a magnetic field strength of 9.4 T. *In vitro* results demonstrated the ability of continuous oxygen evolution by HA-MnO@MSN. After intratumoral administration of HA-MnO@MSN to an HCT116 xenograft mouse model, T_1_ weighted MRI contrast was observed after 5 h postinjection and retained up to 48 h. In addition, *in vivo* photoacoustic imaging of HA-MnO@MSN demonstrated an increase in the tumor oxygen saturation over time after i. t. administration. Thus, the core–shell nanoparticles developed in this study could be helpful in tumor-targeted T_1_ MR imaging and oxygen modulation.

## Graphical Abstract

The hybrid nanoplatform that harnesses the tumor microenvironment factors for dual targeted magnetic resonance imaging and assessment of tumor oxygen status by photoacoustic imaging.

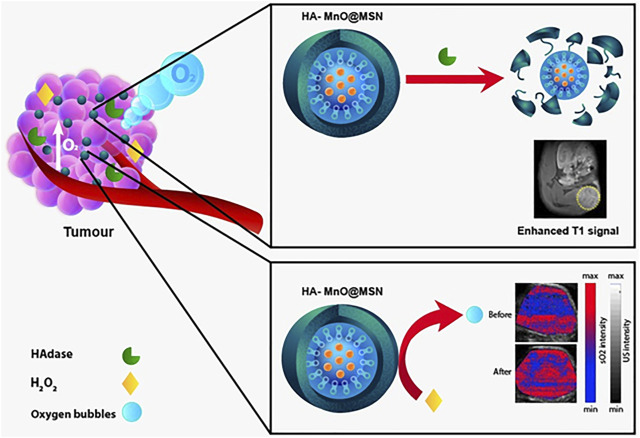

## Introduction

The working principle of magnetic resonance imaging (MRI) is based on protons’ absorption and re-emission of radio waves under a magnetic field. In brief, after irradiation with radio waves of a suitable frequency, protons in the presence of a magnetic field flip and relax back to their equilibrium depending on their physiochemical environment, generating an MRI signal. Owing to their high spatial resolution, excellent soft-tissue contrast, and good patient compliance, MRI holds great promise in diagnostic and clinical imaging (Stark and Bradley, 1999; Gibby, 2005; Sosnovik and Weissleder, 2007). Nevertheless, the low sensitivity of MRI is associated with the inability to delineate imaging contrast between normal and abnormal tissues. In consequence, several nanoparticle-based contrast agents were developed to address this issue. For instance, superparamagnetic iron oxide nanoparticles (SPION) are used as T_2_ (negative) contrast agents, which reduce T_2_ relaxation times by producing dark T_2_ weighed contrast images and are already utilized in clinical settings (Saini et al., 1987; Anzai et al., 2003; Neuwelt et al., 2004; Lin et al., 2009). However, SPION induces blooming effects when administered and causes a vague distinction of hypointense pathogenic conditions, such as bleeding and calcification, which severely hampers its application (Bulte and Kraitchman, 2004; Wu et al., 2004).

In contrast, gadolinium (Gd) complexes emerged as T_1_ contrast agents that can generate hyperintense regions by increasing the longitudinal relaxation T_1_ time, resulting in T_1_ weighed positive-contrast images (Anderson et al., 2006; Caravan, 2006; Aime et al., 2007; Caravan et al., 2007; Granot et al., 2007). In addition, a manganese ion (Mn^2+^) is utilized as a T_1_ contrast agent in neuroscience to investigate the structure and functions of the brain (Silva et al., 2004). However, when administered in high doses, Mn^2+^ can deposit in the brain, causing neurotoxicity (Sepulveda et al., 2012). As a different approach, manganese oxide (MnO) nanoparticles have been explored as a T_1_ contrast agent for molecular and cellular MR imaging (Gilad et al., 2008). Although they have been shown to offer great promise, weak contrast and the absence of prolonged signal duration remain a critical issue for their use *in vivo* (Yang et al., 2010).

The physical properties of manganese (Mn) ions such as the high spin quantum number, extended longitudinal relaxation times, and fast water exchange rates are comparable to those of gadolinium (Gd) ions, which makes them an efficient T1 MRI contrast agent (Mendonça-Dias et al., 1983). Compared to Gd chelates, MnO synthesized as a nanoformulation allows for further chemical modification (HA-MnO@MSN) and is biogenic. HA coating could be selectively degraded by the enzyme hyaluronidase expressed in tumors to exhibit a targeted MR imaging function. Our MnO nanoparticle (NP) also demonstrated a tumor microenvironment modulation property, through which the tumor resident hydrogen peroxide is converted into oxygen. Such oxygen-enriched tumors are conductive to reactive oxygen species–mediated therapeutic modalities such as photodynamic therapy, radiotherapy, and sonodynamic therapy. Gd-based contrast agents could cause nephrogenic systemic fibrosis, raising severe toxicity concerns. In fact, the FDA issues continued caution, and the American College of Radiology guides patients with acute kidney injury to avoid any Gd-based contrast agents (Perez-Rodriguez et al., 2009; ACR Manual on Contrast Media, 2015). As an alternative, Mn-based materials with MR imaging contrast comparable to Gd-based agents were developed (Anbu et al., 2021). In a recent study by Gale et al. (2018), Mn-PyC3A was developed and demonstrated MRI signal contrast comparable to commercially available Gd-DTPA. The authors evaluated various *in vivo* parameters and implied that Mn-PyC3A could be used for MR angiography in patients with renal complications.

Besides imaging functions, recently, the catalytic functions of nanomaterials have drawn significant attention as tumor microenvironment–responsive oxygen generators (Ding et al., 2020). For instance, manganese ferrite nanomaterials have been shown to alter the tumor microenvironment via the catalytic conversion of H_2_O_2_ to O_2_ (Kim et al., 2017). Such oxygen self-supplying nanomaterials have profound applications in cancer therapy (Zhang et al., 2020). Photoacoustic (PA) imaging combines light and sound to enable the functional imaging of the tumor microenvironment (Liao et al., 2014; Zackrisson et al., 2014; Liu et al., 2015; Bandla et al., 2018; Leng et al., 2019). In PA imaging, excited endogenous chromophores (e.g., hemoglobin and melanin) under pulsed laser irradiation undergo thermoelastic expansion, generating pressure waves that are detected by ultrasound (US) transducers. Several studies have shown that PAI can exploit differences in optical absorption characteristics between oxygenated hemoglobin and deoxygenated hemoglobin to obtain estimates of tumor oxygen saturation (%sO_2_) (Mallidi et al., 2015; Chuang et al., 2020). Taking the advantage of MR and PA imaging techniques, the dual-modality approach will enable us to obtain complementary information on tumor biology. MRI allows us to visualize nanoparticle distribution in the tumor, while PA imaging exhibits the tumor oxygenation saturation status. The strength of this dual-modality approach was explored in this study.

For successful biological applications, nanoparticle-based contrast agents must be highly biocompatible and water-soluble, and their surface should be active for further bioconjugation. This can be achieved by coating with mesoporous silica nanoparticles (MSN), a widely used strategy to functionalize contrast agents for biological applications. MSN is biocompatible and nontoxic, and its surface can be modified for desired applications. Owing to these beneficial properties, the enthusiasm for MSN in biological applications has increased exponentially (Cheng et al., 2009; Tu et al., 2009; Cheng et al., 2011; Li et al., 2015; Liu et al., 2016; Wang et al., 2021a). In addition, nanoparticles with an MSN shell can be accessible by water molecules, which significantly enhance the relaxation of water protons. In this study, we report a core–shell nanoparticle that consists of an MnO core and MSN shell. The MSN shell was further chemically conjugated with hyaluronic acid (HA), a negatively charged, nontoxic, and naturally occurring polysaccharide with extensive biomedical applications, such as tissue engineering, hydrogels, drug delivery, and molecular imaging and therapy (Choi et al., 2009; Choi et al., 2010; Tripodo et al., 2015; Dosio et al., 2016; Yan et al., 2016; Phua et al., 2019; Wang et al., 2021a; Zhou et al., 2021). The nanoplatform design in this study is based on the following considerations: 1) HA provides tumor-targeting; 2) HA facilitates controlled access of water molecules to the MnO core; 3) the generation of O_2_ bubbles in the tumor by catalytic decomposition of H_2_O_2_; and 4) the capability to increase the oxygen saturation in the tumor. The validity of this core–shell nanoparticle as an efficient T_1_ contrast agent and its capability as an oxygen generator was further demonstrated both in our *in vitro* and *in vivo* experiments.

## Materials and Methods

### Materials

The chemicals used include tetraethoxysilane (TEOS), cetyltrimethylammonium bromide (CTAB), methanol, sodium hydroxide (NaOH), manganese chloride tetrahydrate, H_2_O_2_, sodium oleate, n-hexane, chloroform, ethyl acetate, 1-octadecene, HA (M.Wt: 10,000–18,000 Da), (3-aminopropyl)triethoxysilane (APTS), and O-(benzotriazol-1-yl)-N,N,N′,N′-tetramethyluronium hexafluorophosphate (HBTU). All chemicals were purchased from Sigma Chemical Co.

### Method Synthesis of Ultrasmall Manganese Oxide Nanoparticles

The synthesis of MnO nanoparticles is described as follows: 1.24 g of the Mn–oleate complex (2 mmol) was dissolved in 10 g of 1-octadecene. The resulting solution was degassed at 70°C for 1 h under vacuum and heated to 300°C with vigorous stirring. The reaction mixture was maintained at this temperature for 1 h to induce sufficient growth. The solution was then cooled to room temperature, and 20 ml of hexane was added to improve the dispersibility of the nanoparticles, followed by adding 80 ml of acetone to precipitate the nanoparticles. The precipitate was obtained by centrifugation. The above purification procedure was repeated two more times to remove the excess surfactant and solvent.

### Synthesis of Mesoporous Silica–Encased MnO

MnO@MSN was prepared using the following procedure. The MnO nanoparticles stabilized with oleic amine were dispersed in chloroform at a concentration of 12.8 mg Mn/ml. Next, typical mesoporous silica coating onto MnO nanoparticles was performed using a sol–gel reaction of TEOS in an aqueous solution containing CTAB and MnO nanoparticles stabilized with the oleic amine. First, 1 ml of MnO nanoparticles in chloroform was poured into 5 ml of 0.05-M aqueous CTAB solution, and the resulting solution was stirred for 1 h, forming an oil-in-water microemulsion. The mixture was then heated to 70**°**C to evaporate chloroform. Next, the resulting transparent solution of MnO/CTAB was added to a mixture of 40 ml of water and 1.4 ml of 2-M NaOH solution, and the mixture was heated to 60**°**C. Then, 0.25 ml of TEOS and 1.8 ml of ethylacetate were added to the reaction solution in sequence, and the reaction was continued for 2 h. The washing steps for MnO@MSN nanoparticles with ethanol were performed to remove unreacted species, and then, the nanoparticles were redispersed in 5 ml of ethanol.

### Synthesis of Hyaluronic Acid–Coated MnO@MSN

For HA coating, we modified the outer surface of MnO@MSN with primary amine groups (MnO@MSN-NH_2_) using APTS. Then, 20 mg of HA and 30 mg of HBTU were added to 1 mg of MnO@MSN-NH_2_ in the phosphate buffered saline solution. The mixture was stirred at room temperature for 4 h, and then, the resulting HA-MnO@MSN was collected and washed with ethanol and water using a centrifuge (12,000 rpm × 3).

### Characterization

The morphology of the samples was characterized using a transmission electron microscope (TEM) (Hitachi, H-7650), operating at an accelerated voltage of 80 kV. Fourier transform infrared spectroscopy (FTIR) was recorded on a Nicolet 550 spectrometer using KBr pellets (approximately 1 mg of the sample was pressed with 300 mg KBr). ZetaSizer Nano was used to measure the hydrodynamic size of the nanoparticles. MRI acquisitions were performed on a 9.4 T magnet (Bruker-Biospin, Billerica, MA, United States) using a 35-mm volume quad-coil (Bruker-Biospin, Billerica, MA, United States). Mn concentrations were based on the molar concentration of manganese atoms measured using ICP-MS (Perkin Elmer Elan 6100).

### Magnetic Resonance Imaging Parameters for *in vitro* Relaxivity Measurements

T_1_ relaxation times were calculated using a RAREVTR inversion recovery sequence. Ten experiments were performed with inversion times (TR) ranging from 150 to 10,000 ms, TE = 9.8 ms, matrix = 128 × 128, FOV = 0.30 mm, slice thickness = 0.30 mm, and NEX = 2. The specific relaxivity (*r*_1_) of the MnO nanoparticles was measured as follows. Each sample was prepared in five different concentrations, and T_1_ values were measured for each concentration, which was then used for *r*_1_ calculations. Relaxivity was determined from the slope of concentration-dependent T_1_ changes.

### *In vitro* US Imaging

*In vitro* US imaging of MnO@MSN and HA-MnO@MSN was performed in 200-µM H_2_O_2_ solution. An agarose gel (3%, w/v) phantom was prepared using a 500 μl Eppendorf tube, and the tube was removed after the phantom gel had cooled. Nanoparticles in 200-µM H_2_O_2_ solutions (1 mg/ml) were prepared and placed in the agarose phantom, and the change in US intensity for each sample was measured up to 30 min using a homemade 128-channel high-frequency US platform (Vantage 128, Verasonics Inc., Washington, DC, United States). The entire US system was controlled using a custom-developed graphical user interface (GUI) based on MATLAB^®^ (R2007a, MathWorks Inc., Natick, MA, United States). The US signals were acquired using a high-frequency 18.5-MHz US transducer (L22-14v, Verasonics Inc., Washington, DC, United States).

### Oxygen Evolution and Quenching of H_2_O_2_

To investigate the ability of oxygen-evolving property, 1 mg of MnO@MSN and HA-MnO@MSN were incubated with 200 μM of H_2_O_2_, and then, the O_2_ concentration was measured using a dissolved oxygen meter (OX10, Unisense Instruments, Denmark). For the quenching experiments, HA-MnO@MSN (1 mg/ml) was added with H_2_O_2_ (10 mM) to initiate the reaction. The residual concentration of H_2_O_2_ was determined over time by measuring the absorbance of H_2_O_2_ at 210 nm. The continuous catalytic effect was verified by repetitive addition of 10 mM of H_2_O_2_ to HA-MnO@MSN (1 mg/ml) solution, followed by determining the concentration by measuring the absorbance.

### *In vivo* Tumor Oxygen Saturation Measurements by PA Imaging

*In vivo* tumor oxygenation saturation (sO_2_) measurements were performed using a PA imaging system. For this purpose, three HCT116 tumor–bearing mice (size ranging from 600 to 750 mm^3^) from the National Laboratory Animal Center, Taiwan, were deeply anesthetized with isoflurane (1–4%) using an inhalation device and placed on a heating pad. Note that these three mice were used for proof-of-concept experiments in this study. Then, 500 µg of HA-MnO@MSN was injected intratumorally into the subcutaneous HCT116 tumor–bearing mice. The baseline image was acquired preinjection and following measurements at various time points postinjection. A 128-channel Verasonics high-frequency US platform (Vantage 128, Verasonics Inc., Washington, DC, United States) was employed for dual-modality imaging (both PA imaging and US imaging). The entire PA system was controlled using a custom-developed GUI based on MATLAB^®^ (R2007a, MathWorks Inc., Natick, MA, United States). In order to operate the system in the PA mode, laser excitation and data acquisition were synchronized using triggering. The excitation laser was a compact Nd:YAG-laser system with an integrated tunable optical parametric oscillator (OPO, SpitLight 600 OPO, InnoLas Laser GmbH, Krailling, Germany). The OPO generates approximately 7-ns duration pulses at a 20-Hz repetition rate with tunable wavelengths from 680 to 2,400 nm. The PA signals were acquired using a high-frequency 18.5-MHz US transducer (L22-14v, Verasonics Inc., Washington, DC, United States). This transducer has a −6-dB fractional bandwidth of 67% and 128 active elements. The acoustic waves were received, reconstructed, and displayed on a computer screen at a frame rate of 20 frames per second. The American National Standards Institute safety limit is 20 mJ/cm^2^, and the incident energy density on the sample surface during PA imaging was estimated to be approximately 12 mJ/cm^2^, which is within the safety limit. The sO_2_ around the tumor was measured via the differential optical absorption of oxygenated and deoxygenated hemoglobin at different wavelengths of 850 and 750 nm, respectively. To facilitate the comparison of sO_2_ patterns in different groups, the regions of interest in the tumor were employed in the proximity of the HA-MnO@MSN injection site and identified using US imaging. PA B-scans of mice generating averagely oxygenated and deoxygenated hemoglobin signals were analyzed using custom-developed software based on MATLAB^®^ (R2007a, The MathWorks, United States), and sO_2_ is defined as sO_2_ = [HbO_2_]/[HbO_2_] + [Hb]. A customized, precision 3D translation stage with motorized x-, y-, and *z*-axes was used to control the transducer to obtain A-scan, B-scan (i.e., two-dimensional; one axis is the lateral scanning distance, and the other axis is the imaging depth), and C-scan (i.e., three-dimensional) images. For *in vivo* imaging, the PA probe was immersed in an acrylic water tank with a rectangular cutout at the bottom serving as an imaging window. The cutout was sealed with a thin polyethylene film of 15-μm thickness. US gel (POC Medical, Inc., Zhongli City, Taiwan) or an agarose pad was then used to provide a coupling interface between the imaging window and the animal.

### *In vivo* Magnetic Resonance Imaging of HA-MnO@MSN in an HCT116 Tumor Model

For *in vivo* MR imaging studies, cultured HCT116 cancer cells (2 × 10^6^ cells) were injected into the right thigh regions of mice (*n* = 3) to establish the tumors. Note that these three mice were used for proof-of-concept experiments in this study. After the tumors developed up to a size of approximately 200 mm^3^, mice were deeply anesthetized with isoflurane (1–4%) using an inhalation device and placed on a heating pad. Then, 500 µg of HA-MnO@MSN was injected intratumorally. MRI acquisitions were performed on a 9.4 T magnet (Bruker-Biospin, Billerica, MA, United States) using a 35-mm volume quad-coil (Bruker-Biospin, Billerica, MA, United States). T1 relaxation times were calculated using a RAREVTR inversion recovery sequence. The baseline image was acquired preinjection and following measurements at various time points postinjection. The MR parameters were set up as TR = 600 ms, TE = 10.5 ms, FOV = 4 × 4 cm, slice thickness = 1 mm, 20 slices, NEX = 8, and matrix size = 256 × 128 recovered to 256 × 256.

## Results

**FIGURE 1:**
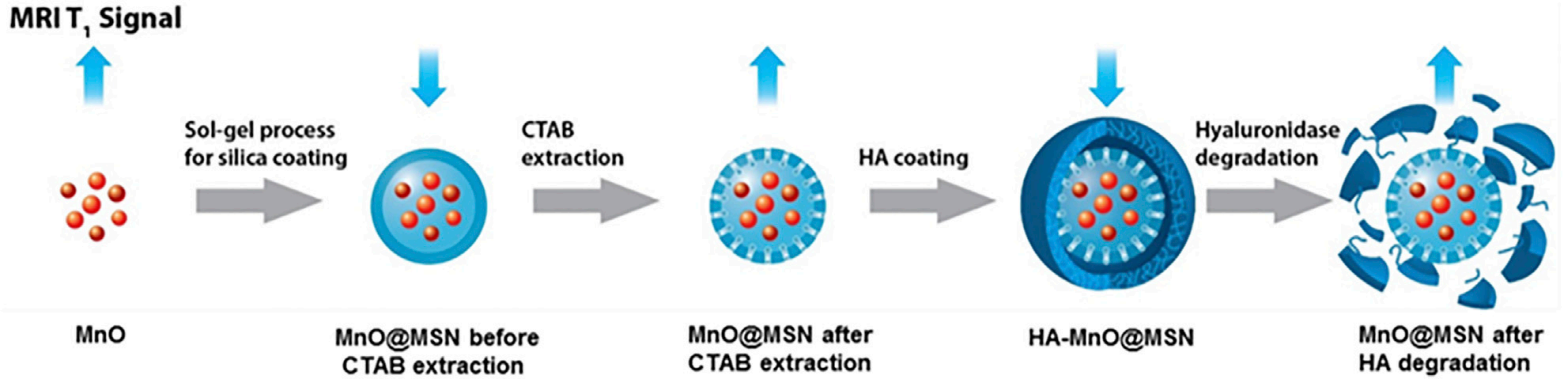
Schematic illustration of the enzyme-responsive and tumor-specific HA MnO@MSN-based T_1_ contrast agent.

In an attempt to develop a hyaluronidase enzyme (HAdase)–sensitive T_1_ contrast agent, HA-conjugated core–shell nanoparticles were prepared. Core–shell nanoparticles consist of an MnO core and HA-MSN as a shell, in which HA was chemically conjugated to the MSN outer surface. A schematic of the concept is presented in Figure 1. Because HA is specifically degraded by HAdase, which is abundant in cancer cells, such nanoparticles may offer potential as carriers for selective cancer imaging and drug delivery.

### Synthesis

**FIGURE 2:**
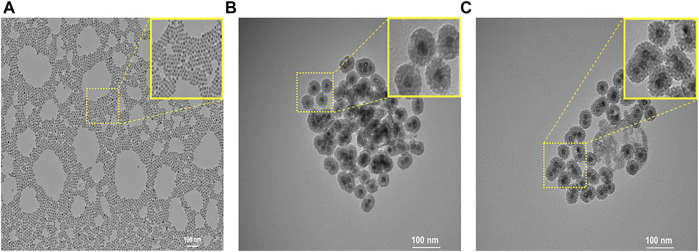
TEM images of **(A)** MnO, **(B)** MnO@MSN, and **(C)** HA-MnO@MSN. Inset shows the magnified image.

Oleic acid–stabilized MnO nanoparticles with an average diameter of 15 nm were synthesized via the thermal decomposition of the manganese–oleate complex (Na et al., 2007). To prepare the MSN shell, a hydrophobic oleic acid–capped MnO nanoparticle was transferred into an aqueous solution using CTAB. Then, a sol–gel-type condensation reaction with TEOS resulted in the formation of an MSN shell. Next, in order to prepare enzyme-responsive nanoparticles, we modified the outer surface of MSN with primary amine using APTS. This is because the repeating unit of HA contains carboxylic acid, which was chemically conjugated to primary amine-functionalized MSN in the presence of HBTU via amide bond formation. At last, CTAB was removed by extraction with acidic ethanol to generate a mesoporous silica shell. Figure 2 shows the TEM images of nanoparticles. MnO nanoparticles were highly monodisperse and spherical in shape, with an average diameter of 15 nm (Figure 2A). The TEM image (Figure 2B) of MnO@MSN shows the MSN coating of MnO with a distinct core–shell morphology, and HA-MnO@MSN particles exhibited a spherical or quasispherical shape with an average diameter of 50 nm (Figure 2C).

**FIGURE 3:**
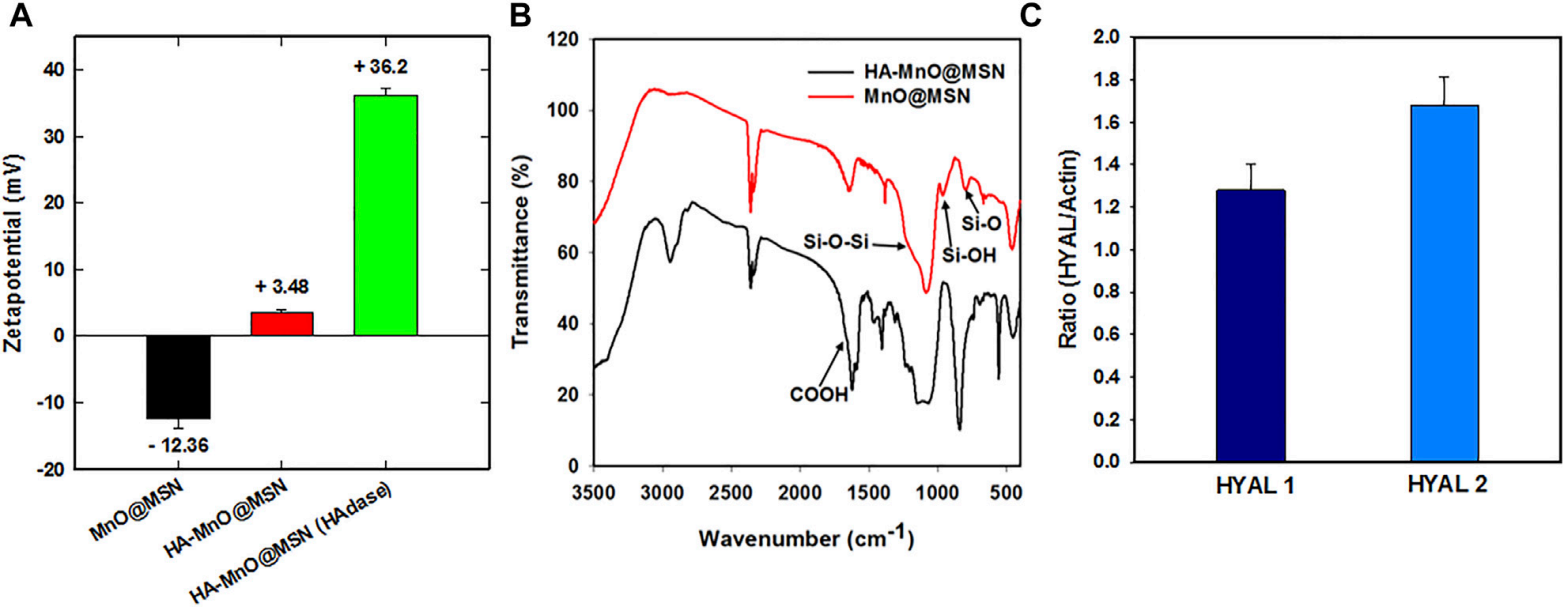
**(A)** Zeta potential, **(B)** FTIR spectra of HA-MnO@MSN and MnO@MSN, and **(C)** distribution of HYAL1 and HYAL2 in HCT116 using western blot analysis (the *p*-value < 0.005).

The surface coverage of HA on MnO@MSN was supported by the zeta potential results (Figure 3A), which increased from −18 (MnO@MSN) to 5 mV. When treated with the enzyme HAdase, the zeta potential of HA-MnO@MSN increased further to 37 mV. This might be due to the digestion of HA by HAdase revealing primary amine groups on the MSN outer surface. Figure 3B shows the FTIR spectra of MnO@MSN and HA-MnO@MSN. From the MnO@MSN spectrum, we can observe the characteristic peak of the silica structure at 760 cm ^−1^ (Si–O stretching), 960 cm ^−1^ (Si–OH stretching), and 1,200 cm ^−1^ (Si–O–Si stretching). After conjugation with HA, the resulting HA-MnO@MSN exhibited similar peaks of silica and a notable sharp peak at 1,620 cm^−1^, which corresponds to the COOH asymmetric stretching of HA, which confirms successful chemical conjugation. Western blot analysis was utilized to study the expression of the HAdase enzyme in HCT116 cancer cells. The analysis showed that HCT116 cells expressed both hyaluronoglucosaminidase 1 (HYAL1) and hyaluronoglucosaminidase 2 (HYAL2) (Figure 3C). HYALl and HYAL2 were present in cell lysate, which indicates that HCT116 cells express and secrete both HAdases.

### Relaxivity Measurements

**FIGURE 4:**
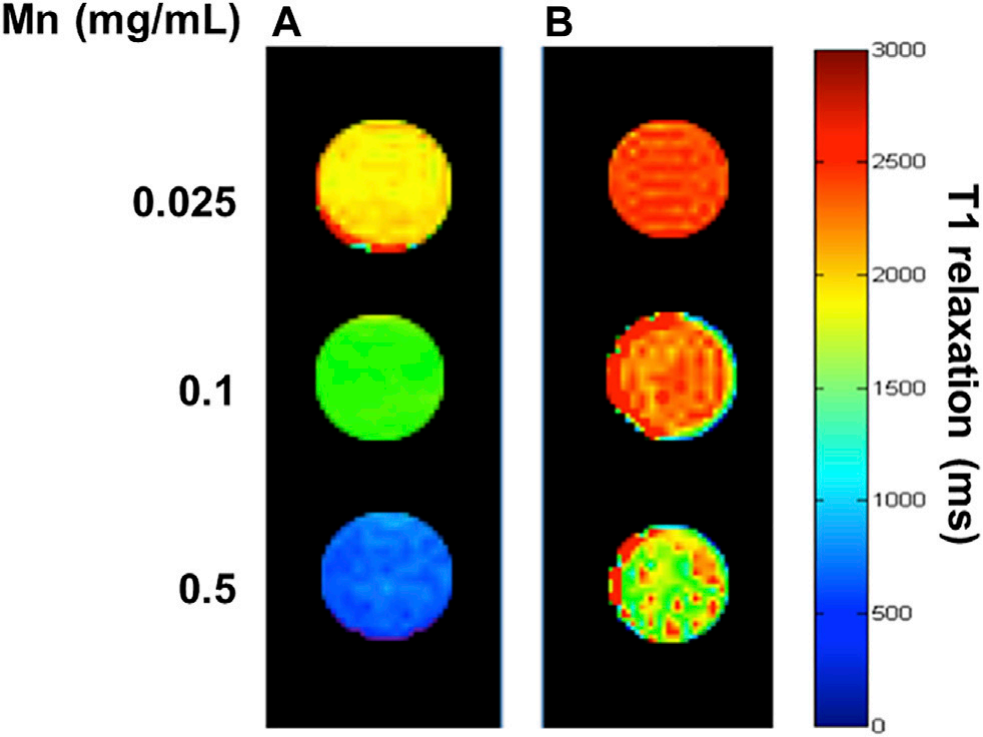
T_1_ map of CTAB-extracted MnO@MSN **(A)** and **(B)** non-CTAB–extracted MnO@MSN at various concentrations suspended in water at 9.4 T.

The primary strategy to increase the r_1_ relaxivity of MnO NP is to increase the availability of water molecules in closer proximity to the magnetic core. Because the major relaxation mechanism constitutes the dipole-dipole coupling between water protons and the manganese ions (Hsu et al., 2016). In the case of the core-shell-structured HA-MnO@MSN in this study, we achieved the r1 relaxivity increase via the structural modifications of the coating to enhance its water permeability. To test the use of MnO@MSN as a T_1_ contrast agent, its longitudinal relaxivity was characterized using a 9.4 T MRI scanner in aqueous suspension. Figures 4A,B show a significant decrease in relaxation time for the incremental MnO concentrations. The molar relaxivity of CTAB-extracted MnO@MSN was determined to be 1.29 mM^−1^s^−1^, which was calculated by measuring the relaxation rate with increasing MnO concentrations. Relaxivity is defined as the change in the relaxation rate of water protons in the presence of contrast agents. The *r*_1_ value of CTAB-extracted MnO@MSN was significantly greater than the values of bare MnO (∼0.28 mM^−1^ s^−1^) and non-CTAB–removed MnO@MSN (∼0.108 mM^−1^ s^−1^). It should be noted that the increase in *r*_1_ value is mainly due to the MSN shell, which is consistent with previous reports (Kim et al., 2007; Kim et al., 2011). MSN allowed optimal access of water molecules through its nanochannels to the MnO core, thus effectively relaxing the nearby water protons. For non-CTAB–removed MnO@MSN with similar MnO concentrations, the presence of CTAB in the MSN nanochannel significantly affected the interaction between the MnO core and water, thus indicating poor T_1_ relaxation.

### Effect of CTAB and HAdase on Relaxivity

**FIGURE 5:**
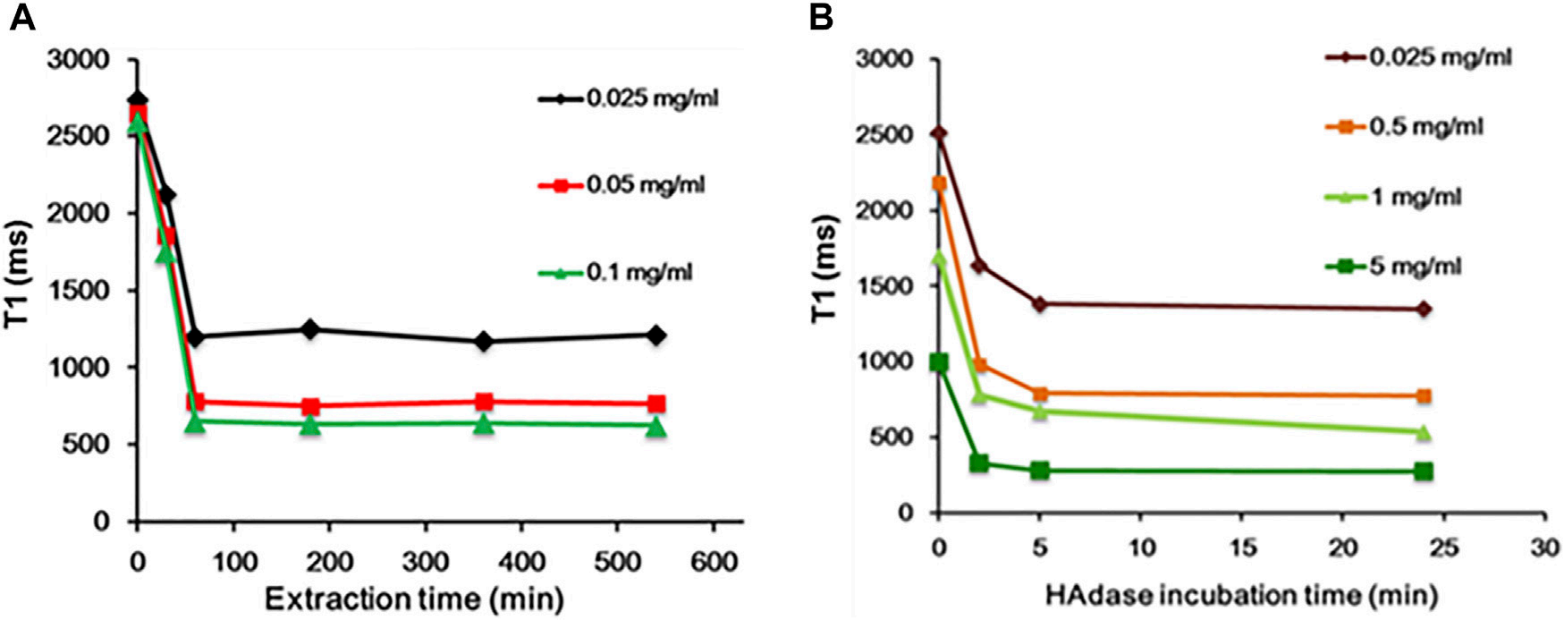
Time-dependent analysis. **(A)** Decrease in T_1_ of MnO@MSN during CTAB extraction and **(B)** decrease in T_1_ of HA-MnO@MSN during incubation with the HAdase enzyme.

In general, CTAB used as a structural directing agent in the synthesis of MSN must be completely removed from nanochannels for successful loading of guest molecules, such as anticancer drugs (Wu et al., 2013). In this study, CTAB was removed by acidic extraction with ethanol for 3 h. The presence of CTAB in MSN nanochannels could act as a barrier to the diffusion of water molecules to the MnO core, which will significantly affect MRI properties. In addition, CTAB is known to induce dose-dependent cytotoxicity (Wang et al., 2008; He et al., 2011; Schachter, 2013), and thus, removal of CTAB is essential for clinical applications. Hence, we evaluated relaxivity at various time intervals during CTAB extraction. As shown in Figure 5A, during CTAB extraction from 0 to 60 min, a steady decrease in the relaxivity of MnO@MSN was observed, followed by little or no significant decrease in the relaxivity up to 9 h. This indicates that CTAB was partially removed in 30 min, followed by the complete removal at 60 min. This result suggests that CTAB removal is crucial for the molar relaxivity of MnO@MSN.

Likewise, the molar relaxivity of HA-MnO@MSN was also investigated in the presence of HAdase (Figure 5B), an enzyme degrading the backbone of HA. Upon incubation with HAdase, the relaxivity of HA-MnO@MSN decreased remarkably, which might be due to the degradation of the HA backbone. This result indicates that HA-MnO@MSN can be used as an enzyme-responsive contrast agent for MR imaging. It is well known that HAdase is abundant in the cytosol of cancer cells. Hence, this unique behavior of HA-MnO@MSN may allow the development of site-specific drug delivery systems for cancer therapy.

Owing to their tunable structures and unique physiochemical properties, manganese oxide nanomaterials (MON) have drawn attention in various biomedical applications such as bioimaging, biosensing, and drug/gene delivery (Ding et al., 2020). Of late, the catalytic activity of these nanomaterials found significant applications as tumor microenvironment–responsive biomaterials. It is well known that the tumor microenvironment is characterized by hypoxia, mild acidity, and elevated production of H_2_O_2_ (Brown and Wilson, 2004). MON has the potential to alter the tumor microenvironment by catalyzing H_2_O_2_ to O_2_, which can be utilized for bioimaging and anticancer therapies.

**FIGURE 6:**
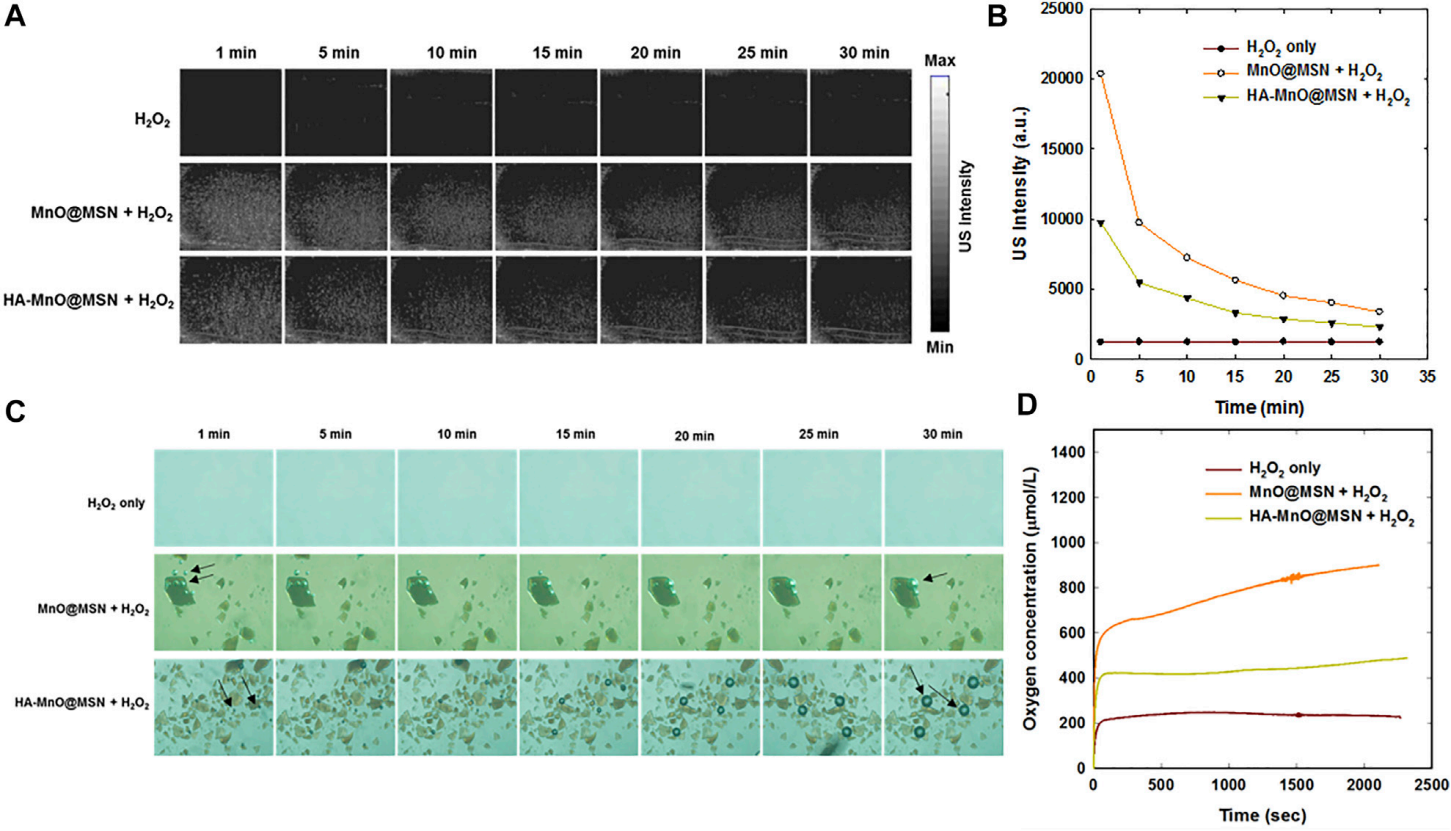
**(A)** Time-dependent *in vitro* US images, **(B)** quantitative analysis of time-dependent US images, **(C)** optical images of oxygen bubble profiles at various time points (arrow indicates oxygen bubbles), and **(D)** evaluation of the oxygen evolution profile measured using an oxygen electrode.

We investigated the generation of oxygen bubbles by HA-MnO@MSN using US imaging. Gas bubbles are excellent contrast agents for US imaging. However, gas- or air-filled bubbles suffer from poor stability *in vivo*, and their inability to target disease constitutes major challenges. In addition, tumor vasculature permeation is difficult because of their size (3–10 µm) (Paefgen et al., 2015). The formation of oxygen bubbles through the catalytic conversion of H_2_O_2_ to O_2_ by HA-MnO@MSN could serve as an excellent US contrast agent due to the ability of gas bubbles to reflect US by generating strong signals. Figure 6A shows the US imaging of H_2_O_2_ alone and NP treated with H_2_O_2_. In the H_2_O_2_-only group, there was no US signal throughout the study because H_2_O_2_ alone could not generate bubbles. Although both HA-MnO@MSN and MnO@MSN showed strong US signal intensities, this was due to the catalytic conversion of H_2_O_2_ to O_2_. The formation of oxygen as nanobubbles or microbubbles was able to strongly reflect US by generating strong signals. Quantitative analysis showed that the US signal generated from HA-MnO@MSN was slightly weaker than that from MnO@MSN, and this might be due to the presence of HA coating delaying the access of H_2_O_2_ to reach the MnO core to start the catalytic reaction (Figure 6B). We observed the direct formation of oxygen bubbles through optical imaging under the same conditions to support US imaging. The formation and growth of oxygen bubbles can be clearly seen in both MnO@MSN and HA-MnO@MSN treated with H_2_O_2_ (Figure 6C).

**FIGURE 7:**
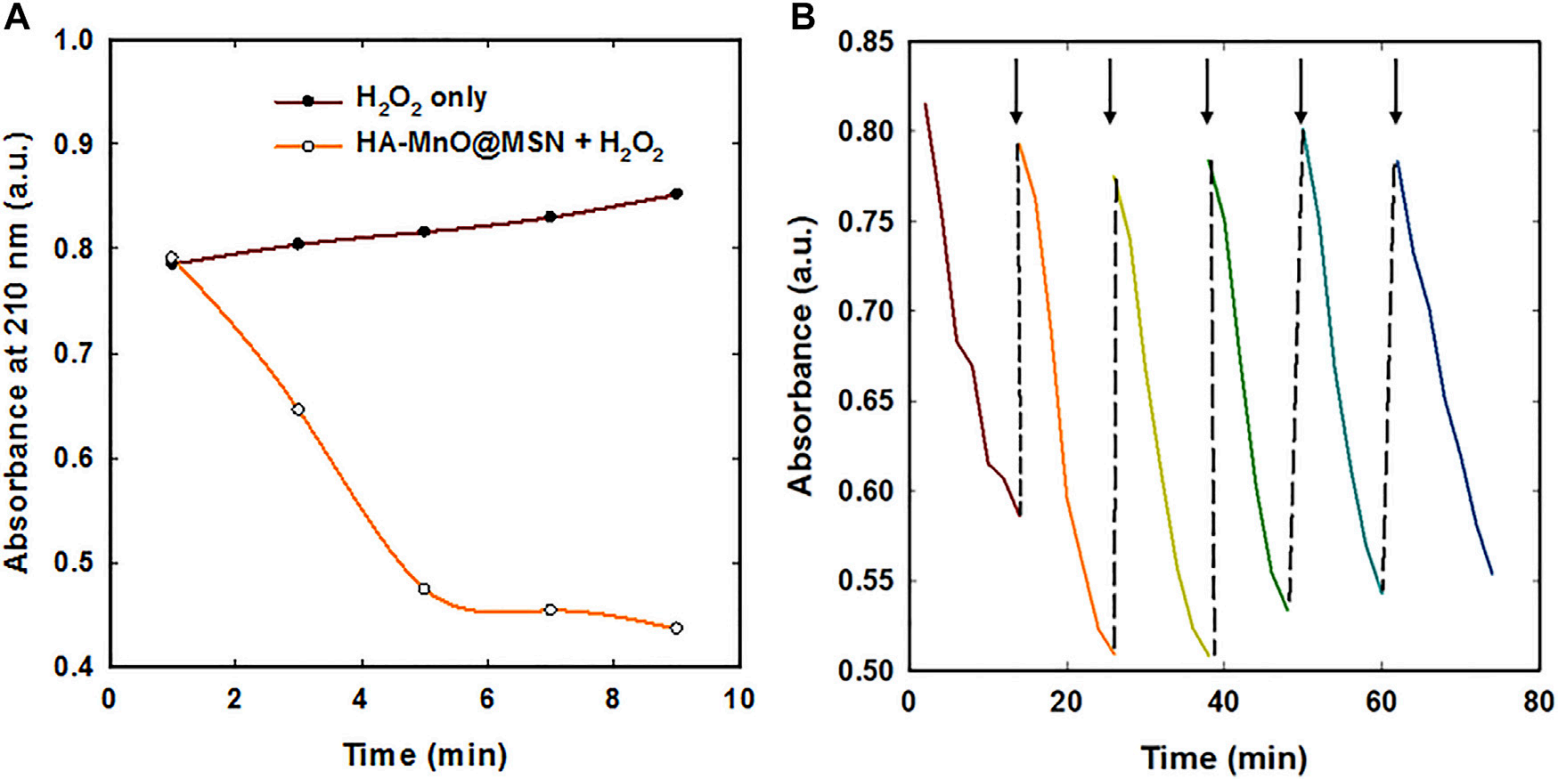
**(A)** H_2_O_2_ quenching over time by HA-MnO@MSN and **(B)** continuous catalytic activity of HA-MnO@MSN by repeated addition of H_2_O_2_ measured by UV–Vis absorption spectra (arrow indicates H_2_O_2_ addition).

We further tested whether our NP can generate a sufficient amount of oxygen at a low H_2_O_2_ concentration using an oxygen electrode. As anticipated, a significant amount of oxygen was generated by both MnO@MSN and HA-MnO@MSN (Figure 6D). Then, we evaluated the catalytic effect of HA-MnO@MSN by measuring residual H_2_O_2_ after the addition of HA-MnO@MSN, and we found that H_2_O_2_ was quenched by HA-MnO@MSN (Figure 7A). One of the important features of HA-MnO@MSN is its capability to continuously generate O_2_. The continuous catalytic activity of the nanoparticles was maintained, even after the repetitive addition of H_2_O_2_ (Figure 7B).

### *In vivo* Tumor Oxygen Modulation by HA-MnO@MSN

Therapeutic approaches such as radiotherapy, photodynamic therapy, and sonodynamic therapy exert their therapeutic effects via copious ROS generation to kill cancer cells (Yang et al., 2019; Wang et al., 2021a). One of the important pitfalls of these therapeutic approaches is the presence of low oxygen tension in the tumor that results in low or partial therapeutic effects, which encourages the residual tumor mass to migrate to various organs, resulting in metastasis. As a consequence, supplemental tumor oxygenation is imperative for better therapeutic outcomes. The rationale for supplemental tumor oxygenation is that the resulting increase in arterial pO_2_ will enhance the diffusion of soluble oxygen into tissues. For instance, carbogen breathing has been shown to improve the oxygenation of both experimental and human tumors. Carbogen is a normobaric high-oxygen-content gas mixture (95% O_2_ with 5% CO_2_ or 98% O_2_ with 2% CO_2_) that increases intravascular oxygen availability, resulting in greater oxygen uptake by tumors (Alonzi et al., 2009). Moreover, various nanomaterials have been recently used to supply oxygen to tumors. These nanomaterials catalytically generate oxygen by consuming hydrogen peroxide in the tumor microenvironment (Zhang et al., 2019; Liu et al., 2020).

**FIGURE 8:**
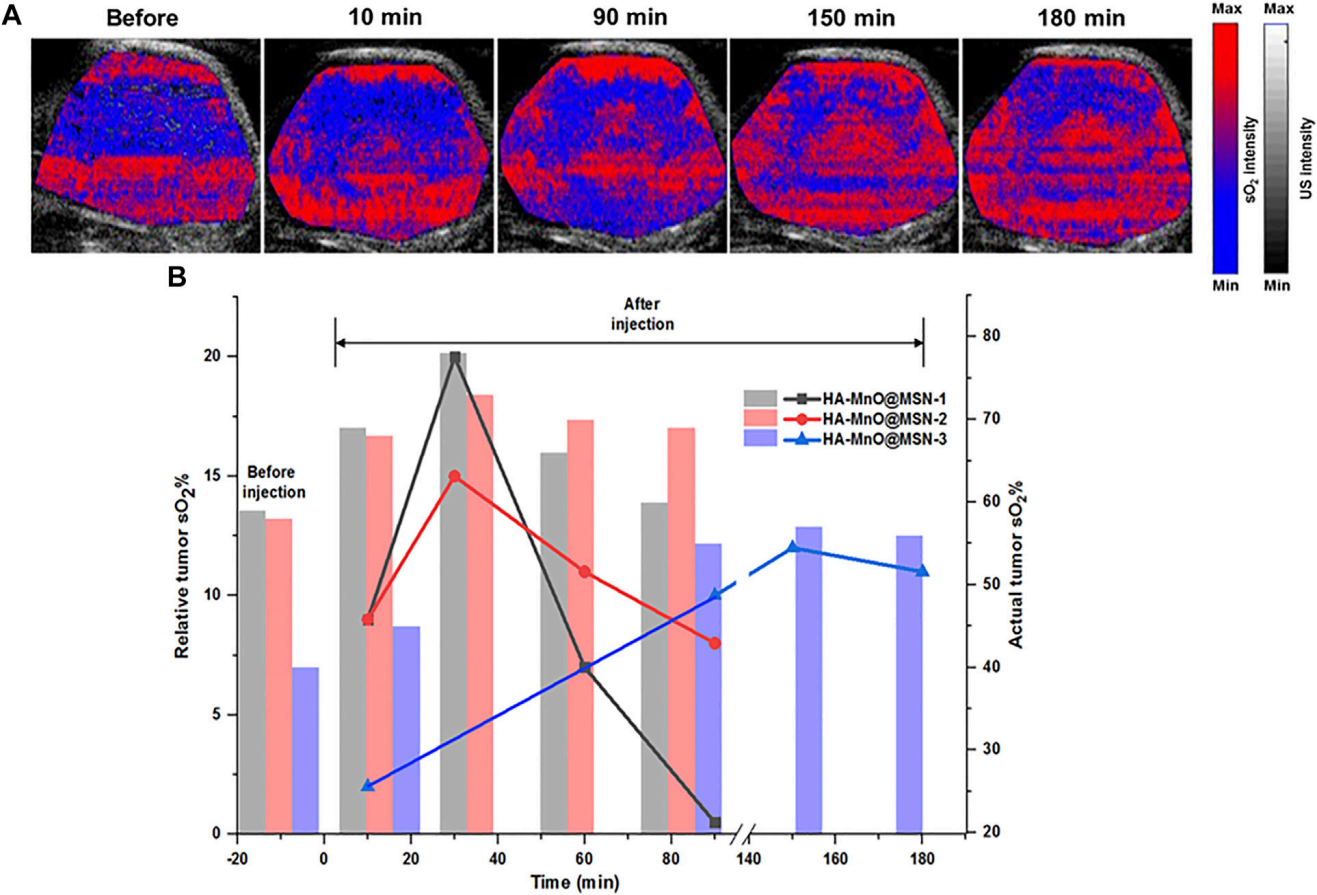
**(A)**Time-dependent PA tumor sO_2_ images of HCT116 tumor–bearing mouse at various time points after i. t. administration of HA-MnO@MSN-3 and **(B)** quantitative PA tumor sO_2_ values at various time points. The bar represents actual tumor sO_2_, and the line represents relative tumor sO_2_ (*n* = 3).

Herein, synthesized MnO@MSN will serve as an oxygen generator by catalase-like activity. From the *in vitro* results, which demonstrated the ability of HA-MnO@MSN to decompose H_2_O_2_, as a consequence, continuous oxygen generation was proved. These results encouraged us to test the capability of our nanoplatform to modulate oxygen saturation in blood inside tumors by PA imaging based on the differential absorption characteristics of oxygenated and deoxygenated hemoglobin. Figure 8A shows the representative sO_2_ map of HCT116 tumors before and after i. t. injection of HA-MnO@MSN 3. The sO_2_ map of the tumor before NP treatment shows the presence of a hypoxia core. After NP injection, the hypoxia level in the tumor core was reduced gradually with an increase in sO_2_ over time due to the catalytic activity of HA-MnO@MSN-3 by converting H_2_O_2_ to O_2_. The observed pattern might be due to the continuous catalytic conversion of H_2_O_2_ by HA-MnO@MSN-3 to generate O_2_ up to 150 min and decreased because of the insufficient levels of H_2_O_2_ for O_2_ generation.

Figure 8B shows the quantitative analysis of relative tumor sO_2_ values from three different experiments after NP injection. HA-MnO@MSN 1 and 2 showed similar tumor sO_2_ trends. For both HA-MnO@MSN 1 and 2, sO_2_ steadily increased from 9% at 10 min to reach a maximum of 20% (actual sO_2_, 69%) (HA-MnO@MSN 1) and 15% (actual sO_2_, 68%) (HA-MnO@MSN 2) at 30 min and then decreased to 0.5 and 8% at 90 min. Next, HA-MnO@MSN 3 demonstrated a unique trend in sO_2_. After i. t. injection, sO_2_ increased to 2% (actual sO_2_, 42%) at 10 min, which is significantly less compared to the sO_2_ values of HA-MnO@MSN 1 and 2, and slowly reached a maximum of 15% (actual sO_2_, 57%) at 150 min, followed by a slight decrease. This relative sO_2_ pattern is inconsistent with actual sO_2_. As previously explained, the presence of a hypoxic core influences the sO_2_ pattern, and tumor hypoxia is characterized by abnormal vasculature, which hinders the supply of nutrients (Brown and Wilson, 2004). Here, HA-MnO@MSN 3 after injection was not able to diffuse in all regions of the tumor because of defective vasculature. Hence, only a few NP had a chance to interact and be digested by HAdase, followed by H_2_O_2_ diffusion to the NP core to generate O_2_ slowly.

### *In vivo* Magnetic Resonance Imaging

**FIGURE 9:**
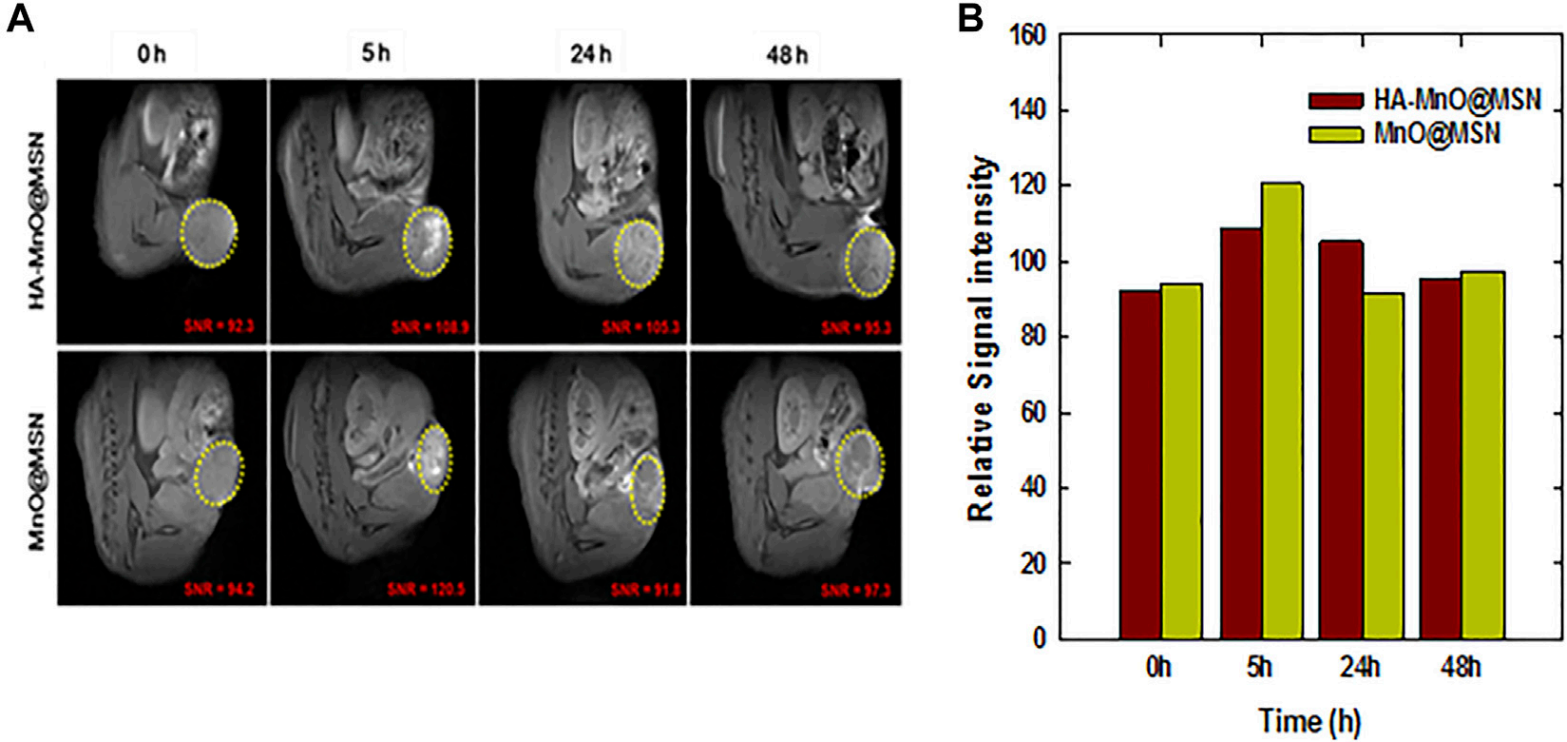
*In vivo* MR imaging of HA-MnO@MSN and MnO@MSN in tumor-bearing mice. **(A)** Time-dependent T_1_ images of nude mice bearing HCT116 tumors after i. t. injection of HA-MnO@MSN and MnO@MSN and **(B)** quantification of HA-MnO@MSN and MnO@MSN in tumor tissue (*n* = 3).

The relaxivity of traditional MnO-based core–shell structures depends on the interaction of MnO with water molecules based on the porosity of the coating material. Herein, HA as a coating material in HA-MnO@MSN would be digested by tumor-specific hyaluronidase to allow the optimal accessibility of the water molecules to the MnO core through nanochannels of MSN. Our *in vitro* results encouraged us to test the potential of HA-MnO@MSN for MR imaging of a tumor, and we administered an intratumoral injection of HA-MnO@MSN and MnO@MSN dispersion to HCT116 human colon tumor-xenograft-bearing nude mice and monitored MR images as a function of time (Figures 9A,B). For HA-MnO@MSN, immediately after injection, we did not observe any contrast enhancement at the tumor site. However, enhancement in T_1_ weighted images was observed at 5 h postinjection. In addition, this contrast enhancement lasted up to 48 h. The T_1_ contrast enhancement was consistently supported by *in vitro* data (Figure 5B), which is due to the time-dependent enzymatic digestion of the HA backbone by HAdase. For MnO@MSN, T_1_ contrast enhancement was observed at 2 h postinjection (data not shown) and slowly decreased after 5 h. This might be a result of the rapid interactions between water molecules and the MnO core due to the lack of HA coating. Thus, MnO@MSN exhibited a rapid but transient T_1_ signal.

**FIGURE 10:**
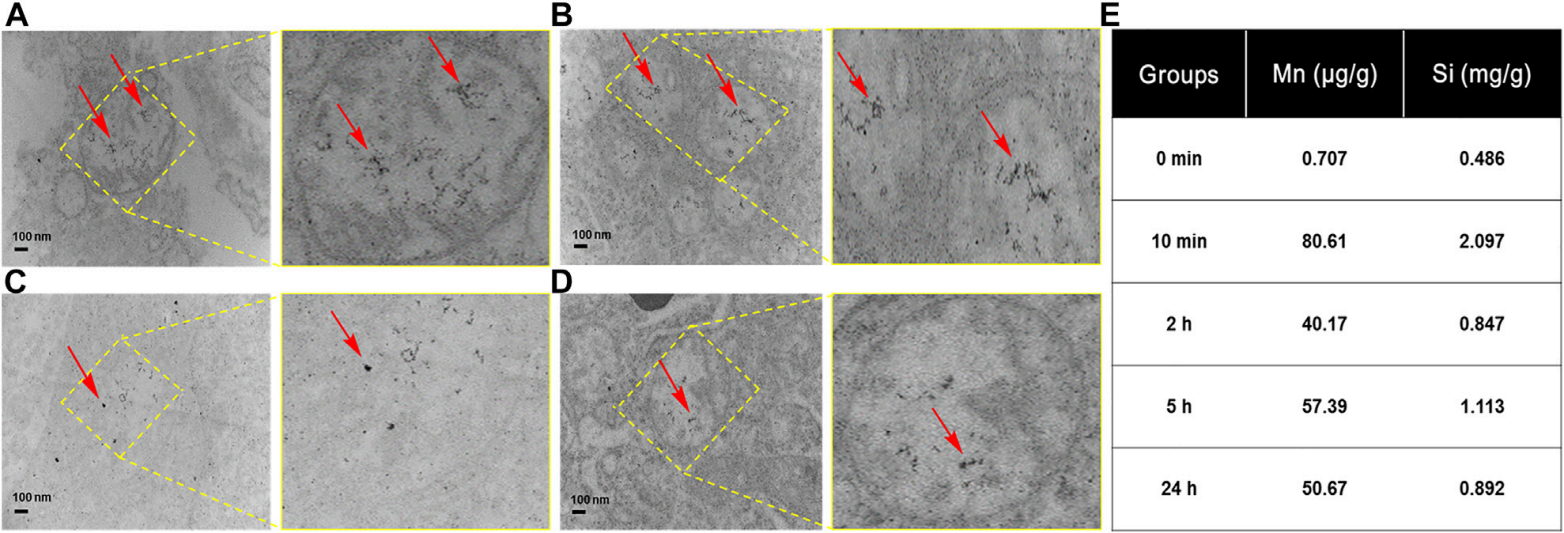
TEM images of tumor tissues obtained from treatment with HA-MnO@MSN at various time intervals with magnified images at **(A)** 10 min, **(B)** 2 h, **(C)** 5 h, **(D)** 24 h, and **(E)** the corresponding ICP-MS of HA-MnO@MSN. The arrows indicate HA-MnO@MSN accumulation in tumor tissues.

To further confirm that HA-MnO@MSN indeed accumulated in the tumor, we used TEM to examine tissue samples obtained from animal models after imaging at various time intervals. As shown in Figures 10A–D, many HA-MnO@MSN (black dots) were clearly seen in tumor samples from 10 min followed by up to 2 h. Then, the slow clearance of nanoparticles from tumor was observed at 24 h. This trend is consistent with ICP-MS data (Figure 10E).

## Discussion

For biomedical applications, we designed HA-coated MnO@MSN NP, which demonstrated various functions such as tumor-specific T1 contrast agent and oxygen modulation. The application of HA as a coating agent offers potential advantages, such as specific binding to cancer cells that overexpress CD44 and digestion by HAdase, found in various metastatic cancers. Hence, HA-based therapeutics have found an integral role in anticancer applications (Huang et al., 2016; Liu et al., 2016; Yan et al., 2016). The controlled degradation of HA by HAdase elicited time-dependent MR imaging functions as a T1 contrast agent as proved here. In particular, we showed here for the first time that MnO is an oxygen generator, which has the potential to modulate oxygen tension in the tumor. In contrast to enzyme catalase, MnO@MSN could be taken up by cells and could have a prolonged *in vivo* half-life. It is well documented that tumor cells express high levels of H_2_O_2_ compared to normal cells. Our nanoplatform’s ability to harness H_2_O_2_ in tumors and oxygenate could instigate the development of smart cancer treatments. *In vivo* PA imaging showed the detailed quantitative assessment of tumor oxygenation in a time-dependent manner after i. t. administration of HA-MnO@MSN. Such NP when combined with ROS-based therapies like PDT will not only improve therapeutic efficacy by enhanced ROS generation but also serve as a marker for treatment efficacy prediction. Furthermore, the use of the MSN shell in this study can be further extended as a host to accommodate guest molecules, such as anticancer drugs or photosensitizers, in its nanochannels with high loading contents. Overall, the unique hyaluronidase-responsive HA-MnO@MSN nanoparticles developed in this study could be used as a tumor-specific theranostic system.

## Conclusion

In summary, we developed HA-MnO@MSN nanoparticles as an MR T_1_ contrast agent, which exhibited higher *r*_1_ relaxivity over bare MnO nanoparticles. HA-MSN coating provides tumor-targeting and allows controlled access of water molecules to the MnO core, resulting in enhanced T_1_ contrast. Our results also demonstrated the continuous oxygen generation ability of HA-MnO@MSN by catalytic reaction both *in vitro* and *in vivo*. These findings demonstrate that the HA-MnO@MSN prepared in this study could be useful for oxygen modulation and targeted MRI.

## Data Availability

The original contributions presented in the study are included in the article/supplementary material. Further inquiries can be directed to the corresponding authors.
